# Comparative transcriptome analysis reveals nicotine metabolism is a critical component for enhancing stress response intensity of innate immunity system in tobacco

**DOI:** 10.3389/fpls.2024.1338169

**Published:** 2024-03-21

**Authors:** Zhongbang Song, Ruixue Wang, Hongbo Zhang, Zhijun Tong, Cheng Yuan, Yong Li, Changjun Huang, Lu Zhao, Yuehu Wang, Yingtong Di, Xueyi Sui

**Affiliations:** ^1^ National Tobacco Genetic Engineering Research Center, Yunnan Academy of Tobacco Agricultural Sciences, Kunming, Yunnan, China; ^2^ College of Resources and Environmental Science, Yunnan Agricultural University, Kunming, Yunnan, China; ^3^ Plant Functional Component Research Center, Tobacco Research Institute of Chinese Academy of Agricultural Sciences, Qingdao, Shandong, China; ^4^ Key Laboratory of Economic Plants and Biotechnology, Kunming Institute of Botany, Chinese Academy of Sciences, Kunming, Yunnan, China; ^5^ State Key Laboratory of Phytochemistry and Plant Resources in West China, Kunming Institute of Botany, Chinese Academy of Sciences, Kunming, China

**Keywords:** ethylene response factor, transcriptomic analysis, nicotine biosynthesis, defensive chemical, environmental fitness, quantitative resistance, *Nicotiana tabacum*

## Abstract

The pyridine alkaloid nicotine acts as one of best-studied plant resistant traits in tobacco. Previous research has shown that *NtERF199* and *NtERF189*, acting as master regulators within the *NIC1* and *NIC2* locus, quantitatively contribute to nicotine accumulation levels in *N. tabacum*. Genome editing-created *Nic1*(*Nterf199*) and *Nic2* (*Nterf189*) double mutant provides an ideal platform for precisely dissecting the defensive role of nicotine and the connection between the nicotine biosynthetic pathway with other putative metabolic networks. Taking this advantage, we performed a comparative transcriptomic analysis to reevaluate the potential physiological and metabolic changes in response to nicotine synthesis defect by comparing the *nic1nic2* and *NIC1NIC2* plants. Our findings revealed that nicotine reduction could systematically diminishes the expression intensities of genes associated with stimulus perception, signal transduction and regulation, as well as secondary metabolic flux. Consequently, this global expression reduction might compromise tobacco adaptions to environmental fitness, herbivore resistances, and plant growth and development. The up-regulation of a novel set of stress-responsive and metabolic pathway genes might signify a newly established metabolic reprogramming to tradeoff the detrimental effect of nicotine loss. These results offer additional compelling evidence regarding nicotine’s critical defensive role in nature and highlights the tight link between nicotine biosynthesis and gene expression levels of quantitative resistance-related genes for better environmental adaptation.

## Introduction

Plant secondary metabolites play a pivotal role as essential bioactive compounds in defending against predators and optimizing plant environmental fitness (Steppuhn et al., 2004). Among these bioactive compounds, alkaloids constitute a class of alkaline organic substances characterized by a cyclic nitrogenous nucleus and are known for their functions in defending against herbivores and pathogen attacks (Tso, 1990; Ashihara et al., 2010). In *Nicotiana* species, 3-pyridyl derivative alkaloids are *in vivo* synthesized and amounts vary in organs with distinct major alkaloid profiles, such as nicotine, nornicotine, and anabasine (Sisson and Saunders, 1982; Saitoh et al., 1985). In cultivated tobacco (*Nicotiana tabacum*), nicotine is the predominant alkaloid and its content in tobacco leaves is recognized as an essential standard for commercial use (Davis and Nielsen, 1999). Pharmaceutically, nicotine binds to neuronal nicotinic acetylcholine receptor subtypes throughout the nervous systems and stimulates midbrain dopamine neuron firing rates and phasic burst firing, and results in neuroadaptations (Tobacco TCPGT, 2008; Wittenberg et al., 2020). Therefore, nicotine serves as the primary addictive substance in combusted tobacco products and e-cigarettes. For public health concerns and the goal of reducing addiction, both the World Health Organization (2015) and the Food and Drug Administration of the United States (2018) have considered mandating standards to minimize nicotine levels in combustible cigarettes to nonaddictive levels (Lewis et al., 2020). The recommended threshold of non-addiction nicotine in the tobacco filler is set below 0.04% (World Health Organization, 2015).

In *N. tabacum*, nicotine is exclusively synthesized in root tissues and subsequently translocated to aerial organs via the xylem system, and accumulates in leaf cells (Dawson, 1941; Thurston et al., 1966). The nicotine molecule is characterized by the presence of a pyrrolidine ring and a pyridine ring. The nicotine content in commercial tobacco cultivars typically ranges between 2% and 4% of the total dry weight basis (Saitoh et al., 1985) and exhibits significant variability influenced by genetic factors, environmental conditions, and agronomic practices (Tso, 1990; Bush et al., 1993). Genetic studies have revealed that alkaloid levels in cultivated tobacco are primarily regulated by two loci, *NIC1* and *NIC2* (Legg et al., 1969 and Legg and Collins, 1971). Among these loci, *NIC1* exerts a 2.4 times stronger effect in determining nicotine and associated alkaloid levels when compared to *NIC2* (Legg and Collins, 1971). Both *NIC1* and *NIC2* loci consist of tandem sets of homologous *ERF* transcription factors located on chromosomes 7 and 19 within the tobacco genome (Shoji et al., 2010; Kajikawa et al., 2017; Sui et al., 2020). The recessive alleles of *Nic1* and Nic2 in nicotine mutants have been attributed to chromosomal deletions (Shoji et al., 2010; Kajikawa et al., 2017) and potentially chromosomal deletion-induced structural variations (Sui et al., 2020; Shoji et al., 2022). Two homologous genes, *NtERF199* and *NtERF189*, which act as master transcriptional activators within the *NIC1* and *NIC2 ERF* clusters, have been found to directly bind to the GCC-box element in the promoters of pathway structural genes, thereby coordinately activating their expression (Shoji et al., 2010; Sui et al., 2020). Furthermore, CRISPR/Cas9-based double mutants have exhibited a low-nicotine phenotype through the simultaneous genome editing of *NtERF199* and *NtERF189* (referred to as *Nic1-2* and *Nic2-2*; Hayashi et al., 2020). Natural mutations in *Nic1* and *Nic2* have been associated with pleiotropic effects on leaf yields, chlorophyll content, and harvest timing (Chaplin and Weeks, 1976). However, near-isogenic *Nic1* and *Nic2* lines generated through traditional breeding methods tend to introduce additional variations caused by chromosomal structural changes which are tightly linked to target genes (Shoji et al., 2010; Kajikawa et al., 2017; Sui et al., 2020). This fact inevitably impedes the precise pinpointing of the impact of the defensive resistance conferred by nicotine. In contrast, the *Nic1-2* and *Nic2-2* double mutants exhibited no abnormalities in growth and development under experimental conditions (Hayashi et al., 2020; Shoji et al., 2023). Therefore, these double mutants provide an ideal system to evaluate the putative defense trait of nicotine in nature. Since they differ only in the loss of function of *NtERF199* and *NtERF189* while otherwise remaining genetically identical (Bergelson and Purrington, 1996; Steppuhn et al., 2004). Taking this advantage, it is interesting to investigate the correlation between plant innate immunity system and the defensive trait of nicotine in tobacco.

In the present study, we conducted a comprehensive comparative transcriptome analysis between wild-type tobacco (*NIC1NIC2*) and low-nicotine lines (*Nic1-2Nic2-2*) that share an identical genomic background. This transcriptomic profiling spanned the entire spectrum of tobacco growth and encompassed early stages in response to topping. The transcriptome analysis provided compelling evidence that nicotine reduction leads to differentially transcriptomic and metabolic alterations at each of the investigated growth stages. The differentially expressed genes identified are intricately linked to various aspects of the plant innate immunity system, including environmental stimulus perception, signal transduction cascade, defensive response, transcriptional regulation, as well as secondary metabolite biosynthesis. In summary, our findings demonstrate that loss-of-nicotine leads to a comprehensive reduction in the expression of a diverse array of genes associated with environmental adaptability, herbivore resistance, and various facets of plant growth and development. Notably, we also observed the up-regulation of a novel set of stress-responsive and metabolic pathway genes which might signify a newly metabolic reprogramming strategy to trade-off the detrimental effects of nicotine loss and compensate overall environmental fitness.

## Materials and methods

### Experimental materials and RNA extraction

Flue-cured tobacco variety Yunyan87 (WT) and *Nic1-2Nic2-2* lines (Hayashi et al., 2020) with identical genomic background were cultivated under standard greenhouse condition at the Yanhe Tobacco Experimental Station in Yuxi, Yunnan province. Seedlings were transplant into plastic pots (33 cm × 27 cm) with cultural media and managed following routine greenhouse practices. To comprehensively capture the transcriptomic differences throughout the different representative growth periods between WT and *Nic1-2Nic2-2*, three vegetative growth stages were selected, including the seedling stage (SD), rosette stage (RS), and before topping stage (BT). In the SD, the typical seedlings usually have thick leaves with relatively large leaf area and enhanced photosynthetic capability. The plantlet also has newly established root system and transport tissue with strong absorption capabilities. The RS refers to the period from seedling transplant to a tobacco plant with 30~35cm in height and 12-16 unfolded leaves. These leaves horizontally developed with the width as twice as the height of the plant. This stage signifies the peak of root growth after tobacco transplant and the underground growth outweighs above-ground growth, with a substantial development of lateral and fine roots. The BT stage extends from plant rosette to the emergence of flower buds and is characterized by vigorous vegetative growth accompanied by flower bud differentiation. During this period, the lateral and fine roots continue to grow, and adventitious roots are formed abundantly. The stem elongates rapidly and the leaves are enlarged and overlapped, spreading out in a trumpet shape. Importantly, the emergence of the flower buds in a tobacco plant signals a transition from a vegetative growth stage to a reproductive growth stage. Flower buds must be removed by topping practice to allow the plant to reach its full yield and improved quality at harvest. After topping (T) practices, different plant phytohormone signaling cascades could be triggered within tobacco plant, which in turn activates their downstream gene expression and initiates the accumulation of diverse array of secondary metabolites. Therefore, leaves from the two groups were also collected at different time points (0.5 h, 1 h, 2 h, 4 h, and 6 h) after topping (T05, T1, T2, T4, and T6) for transcriptomic sequencing and/or gene expression validation. Both WT and *Nic1-2Nic2-2* plants with similar physiological status were selected for sampling. Middle leaves were gently excised using a surgical scissor and frozen immediately in liquid nitrogen and stored in -80°C for RNA extraction. For RNA extraction, the frozen leaf tissues of all seven sampling stages were ground into powder, and total RNA were extracted using RNeasy Plant Mini Kit (Qiagen, USA) following the manufacturer’s guidelines.

### Library preparation and transcriptome sequencing

For RNA sequencing library construction, 2 µg of total RNA was employed. The quality of these RNA samples was assessed using the Agilent 2100 Bioanalyzer (Agilent Technologies, USA). Samples with RNA integrity number (RIN) ≥ 8 were proceeded for library preparation. The library construction was performed using TruSeq^®^ RNA Sample Preparation Kit (Illumina, USA) according to the manufacturer’s protocol. Sequencing reaction was conducted on both the WT control and *Nic1-2Nic2-2* lines with pair-end run (150bp × 2). The amplified libraries were subsequently sequenced on the MGISEQ-2000 sequencing platform, generating a total of 8 GB of 150-bp paired-end reads per sample. Demultiplexing, adapter and barcode sequences trimming were performed on Trimmomatic v0.32 (Bolger et al., 2014). The quality of the data was verified, and sequencing reads were provided in FASTq format. The transcriptome data has been deposited in the Genome Sequence Archive (Chen et al., 2021) in National Genomics Data Center (CNCB-NGDC Members and Partners, 2022), China National Center for Bioinformation/Beijing Institute of Genomics, Chinese Academy of Science (GSA: CRA014456) that are publicly accessible at https://ngdc.cncb.ac.cn/gsa.

### Read assembling and gene expression quantification

The raw paired-end reads were cleaned through removing adaptor sequences, poly-N, and low-quality sequences (i.e. the percentage of bases of quality value≤ 5 exceeds 50% in the read). In addition, short sequences (< 50bp) were also removed using a custom Perl program. All filtered reads were aligned to the tobacco reference genome (Nitab-v4.5_genome_Scf_Edwards2017.fasta.gz) accessible from Sol Genomics Network (Fernandez-Pozo et al., 2015) using HISAT v2.2.1 (Kim et al., 2019). Gene expression levels were quantified as FPKM (fragments per kilobase of exon per million reads fragments mapped reads) value using Cufflinks software version 1.2.1 (Trapnell et al., 2012). Differential expressed genes (DEGs) were identified using the DESeq R package (v1.18.0) and edgeR package (v 3.24.3) (Robinson et al., 2010). The criteria for identifying DEGs were set at a |log2 fold change (FC)| threshold of ≥ 1 and a corrected p-value (q-value) < 0.05, as previously described (Hu et al., 2021). Pair-wise Pearson correlation coefficients for each transcript were calculated using the RPKM. The Matrix distances for the expression heat-map were computed with Pearson correlations of gene expression values (RPKM) by heatmap.2 function of gplots (version 3.0.1) within the Bioconductor package in R (version 3.2.2) (Wickham, 2009).

### Functional annotation and gene ontology

Gene Ontology (GO) and Kyoto Encyclopedia of Genes and Genomes (KEGG) analyses were conducted to identity the enrichment of DEGs in GO terms and metabolic pathways, respectively. A threshold of a corrected p-value (q-value) of ≤ 0.05 was applied to define significantly enriched GO terms and KEGG pathways. Functional analysis of these DEGs was performed in the KEGG Automatic Annotation Server (KAAS) (http://www.genome.jp/tools/kaas/). The KEGG Orthology (KO) terms assigned to the transcripts and categorized them into KEGG pathways with default parameters. Genes identified by DEG analysis were subjected to a GO enrichment analysis using clusterProfiler (version 4.4.2) package (Yu et al., 2012a).

### Quantitative real-time PCR analysis

The qRT-PCR experiments were conducted in a 20 ml reaction volume using the LC480 system (Roche, USA) with three biological replicates. The tobacco Actin gene (accession No.: X63603) was used as an internal standard for normalization, and SYBR Green served as the fluorochrome. A three-step PCR process was performed with a pre-denaturation at 95°C for 30 s, followed by 45 cycles of denaturation at 95°C for 15 s, annealing at the optimal temperature of each primer pair for 15 s, and elongation at 72°C for 20 s, and finally for melting point curve analysis (95°C for 5 s, 70°C for 1 min and 95°C for 15 s) to test amplicon specificity. Relative quantification of gene expression levels was calculated using 2^-△△Ct^ method. Sequence information of the qRT-PCR primers are listed in **Supplementary Table 8**.

### Chemical quantification

In total of 2.0 kilograms of flue-cured middle leaves from both wild-type and *Nic1-2Nic2-2* transgenic tobacco plants were collected for chemical measurements. The contents of six carbohydrate compounds (total sugar, reducing sugar, fructose, starch, glucose, and saccharose) and total nitrogen from the two groups were measured according to Tobacco and Tobacco Products-Determination YC/T 159-2019, YC/T 216-2013, YC/T 251-2008, and YC/T 161-2002, respectively. The contents of total amino acid and carotenoid in each leaf sample were quantified using ninhydrin color reaction and colorimetry as previously described (Wei et al., 2006; Li et al., 2010).

### *Myzus persicae* performance and feeding preference

2-weeks-old seedlings were transplanted individually into plastic pots (10 cm × 7.5 cm) with cultural media at 25°C under 16h artificial light in the growth chamber (Percival Scientific, USA). For analysis of feeding preference, newly enclosed *M. persicae* larvae were placed on the newly developing leaf of 4-week-old WT and *Nic1-2Nic2-2* seedling plants (5 and 5) and allowed to feed for 15 days. The feeding preference of *M. persicae* was determined by the larval mass on the surface of the seedling leaves.

### Agrobacterium infiltration

For transient expression assays, plasmids encoding the TMV-Cg CP protein was introduced into Agrobacterium strain EHA105. The bacteria were grown overnight at 25°C and diluted to an 0.5 A600 density with a 1-mL needleless syringe before infiltration into 4-week-old WT and *Nic1-2Nic2-2* seedling (at the 5 to 8 leaf stage) leaves immediately above the cotyledon (Li et al., 2023). Infiltrated seedling plants were grown in cultural media in an environmentally controlled growth chamber (Percival Scientific, USA). At least 3 seedling plants were used for each experiment and all experiments were repeated three times.

### Statistical analysis

All data are presented as the mean ± standard deviation (SD). Statistical analyses were conducted using a one-way ANOVA model, followed by Student’s t-tests for multiple group comparisons. Significance was determined with unpaired p-values ≤ 0.05 were considered statistically significant.

## Results

### RNA−seq analysis reveals differential gene expression between WT plants and *Nic1-2Nic2-2* lines

Transcriptomic analysis was conducted to comprehensively assess the impact of *NtERF189/199* knockdown on gene expression across three vegetative growing stages and four after-topping time stages. A total of 42 samples, consisting of three biological replicates for both the WT control and *Nic1-2Nic2-2* lines, were utilized for library preparation and subsequent RNA sequencing. Initial filtering of low-quality sequencing reads was performed using FastQC (Andrews, 2010), resulting in a total of 1,582 million (M) clean reads for the WT samples and 1,600 million (M) for the *Nic1-2Nic2-2* samples. On average, each sequencing library contained more than 56 M reads with high-quality base scores (**Supplementary Table 1**). All clean reads from each library were successfully mapped to the tobacco reference genome sequence (Edwards et al., 2017) with genome mapping rates ranging from 91.19% to 96.99% and over 88% of the clean reads mapped to unique genomic positions (**Supplementary Table 2**).

### Nicotine deficient significantly changes gene expression patterns in the *Nic1-2Nic2-2* leaves at different growth stages and response to topping

**Figure 1:**
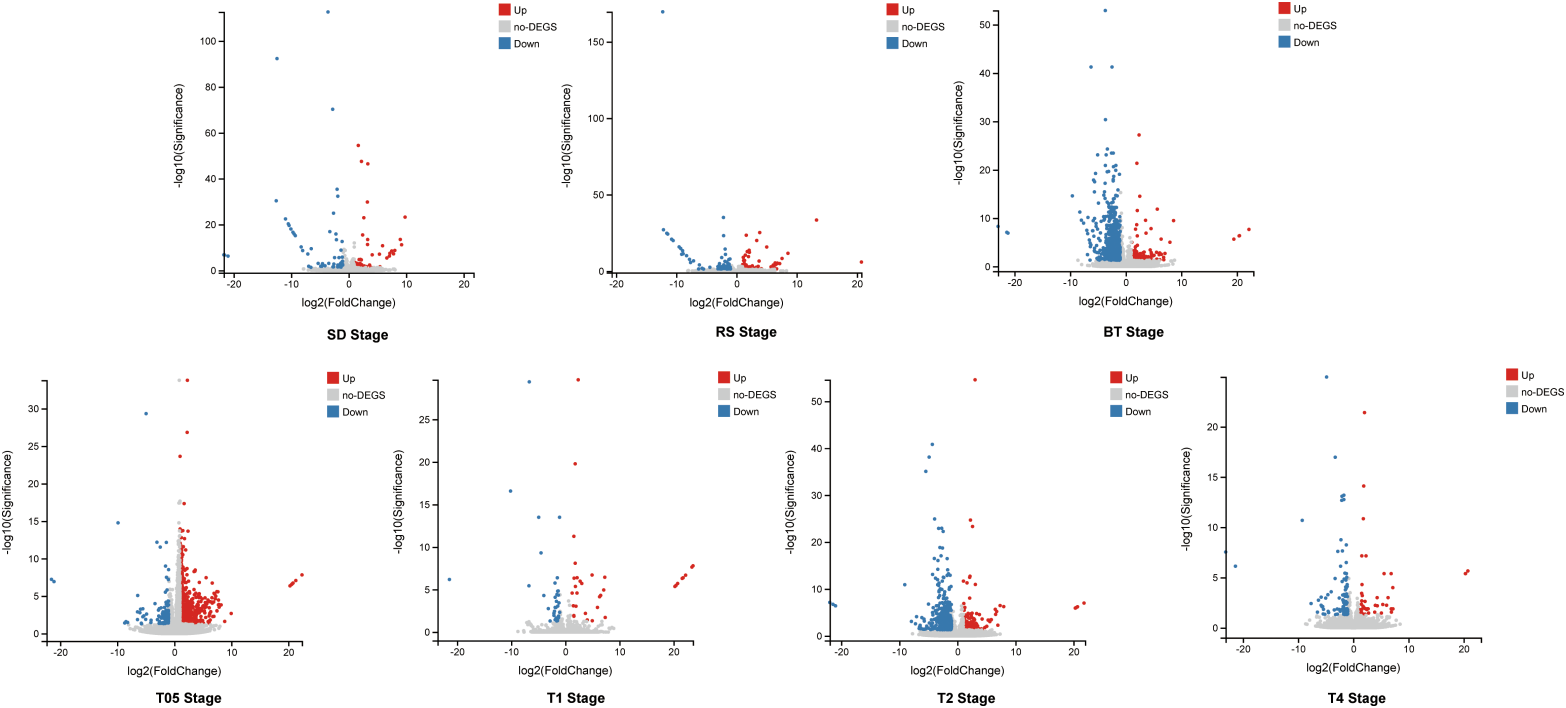
Differential expression analysis of leaf transcriptomes from wild-type and *Nic1-2Nic2-2* transgenic tobaccos at different growing stages. Volcano plot identified DEGs in the transcriptomes of seedling stage (SD), rosette stage (RS), before topping stage (BT), and after topping stages (T05, T1, T2, T4), respectively. The value of |log2 fold changes (FC)|≥1 and an corrected *p*-value (*q*-value) ≤ 0.05 was used as criteria to select significantly expressed gene. The red dots denote the significantly up-regulated DEGs, and the blue dots denote down-regulated DEGs, and the gray dots denote genes are not significantly expressed.

**Figure 2:**
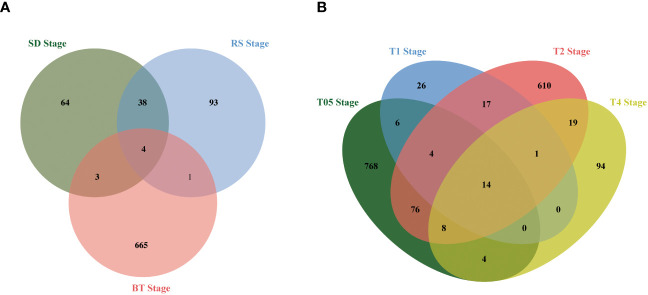
Venn diagram analysis of the DEGs at different growing stages. **(A)** Venn diagram showing the overlap of DEGs among the transcriptomes from the SD stage, RS stage, BT stage, respectively. Compared with the EV control, double knock off of *NtERF189* and *NtERF199* resulted in 109, 136, and 673 DEGs in these three developmental stage transcriptomes. **(B)** Venn diagram showing the overlap of DEGs after topping four time points. Compared with the wild-type control, knocking down of *NtERF189* and *NtERF199* resulted in 880, 68, 749 and 140 DEGs at after topping four-time course points.

To assess the impact of *NtERF189/199* knockdown on tobacco physiological and metabolic changes, a comprehensive transcriptomic analysis was carried out to identify potential gene expression variations. Differential expression analysis was performed to identify DEGs and the quantification of gene expression levels were were quantified using FPKM values. High correlation coefficients (approximately 0.93) were observed across different biological replicates (**Supplementary Tables 3**, **4**). Differentially expressed genes (DEGs) were identified based on FPKM with a threshold set at |log2 fold-change (FC)| ≥ 1 and a false discovery rate (FDR) < 0.05. The knockdown of *NtERF189/199*, in comparison to the WT control, led to significant changes in gene expression patterns during both the vegetative growth stages and after topping practices. In detail, 110 DEGs (55 upregulated, 54 downregulated) were identified at the SD stage, 136 DEGs (58 upregulated, 78 downregulated) at the RS stage, and 673 DEGs (117 upregulated, 556 downregulated) at the BT stage (**Figure 1**). Physical topping also resulted in distinct expression patterns between the WT control and *Nic1-2Nic2-2* transcriptomes at four time points. Specifically, 880 DEGs (737 upregulated, 143 downregulated) were detected at the T0.5 stage, 68 DEGs (33 upregulated, 35 down-regulated) at T1 stage, 749 DEGs (175 upregulated, 574 down-regulated) at T2 stage, and 140 DEGs (45 upregulated, 95 down-regulated) at T4 stage (**Figure 1**; **Supplementary Table 5**). The relationships among the DEGs identified within the three vegetative stages and four after topping stages were illustrated separately using Venn diagrams. As illustrated, the Venn diagram revealed that only 46 DEGs were shared within at least two vegetative growth stages (**Figure 2A**). Similarly, there were 149 DEGs shared by at least two stages among the before and after topping stages (**Figure 2B**). Among the total of 195 DEGs, the downregulated genes were predominantly associated with plant-pathogen interaction and the mitogen-activated protein kinase (MAPK) signaling pathway. In contrast, genes related to starch and sucrose metabolism, amino acid metabolism, heat shock protein (HSP) 20 family genes, and carotenoid biosynthesis were upregulated in *Nic1-2Nic2-2* lines compared to the WT control. The differentially expressed genes for each growth stage are listed in **Supplementary Table 5**.

### Gene ontology enrichment analysis highlights the critical role of nicotine biosynthesis in maintaining typical responses to stress stimuli, secondary metabolite biosynthesis, and regulatory processes

**Figure 3:**
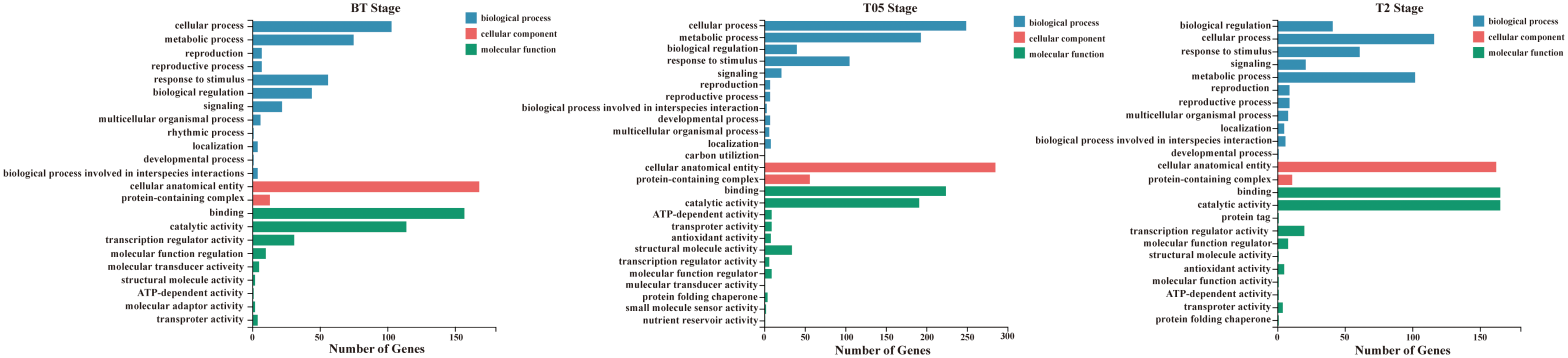
Gene ontology (GO) analysis of three selected growing stage (BT, T05, and T2) transcriptomes. GO term enrichment analysis. Significantly enriched GO terms were selected based on an FDR<0.05. GO terms of the categories of biological processes, cellular components, and molecular functions are depicted in blue, red, and green, respectively.

To assess the impact of *NtERF189/199* knockdown on the physiological status of tobacco, we conducted gene ontology (GO) enrichment analysis to further explore the DEGs across various growth stages. Significantly enriched GO terms for DEGs were determined with a Q-value threshold of ≤ 0.05. Specifically, three key growth stages (BT, T0.5 and T2) were selected for GO enrichment analysis as these results hold particular significance in addressing our research objectives (**Figure 3**). The DEGs were categorized into three ontologies: biological processes, cellular components, and molecular functions.

**Figure 4:**
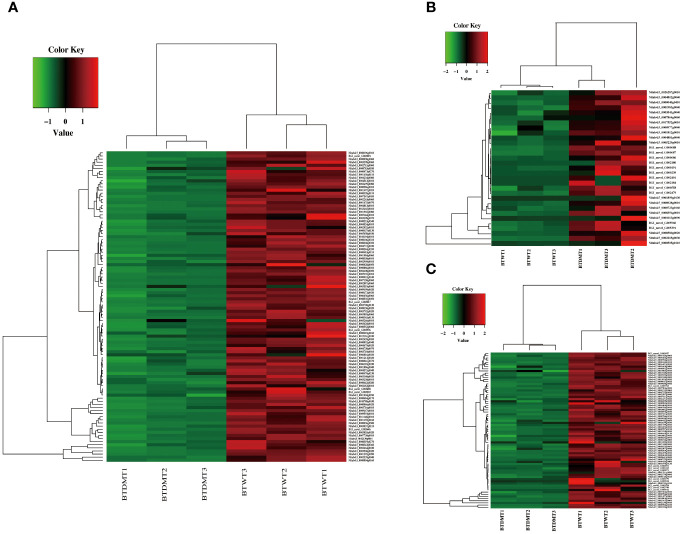
Differential gene expression analysis and hierarchical cluster analysis of the BT stage transcriptomes. **(A)** Heatmap analysis of DEGs based on the KO terms in plant-pathogen interaction, plant MAPK signaling pathway, and plant hormone signal transduction. **(B)** Hierarchical cluster analysis of the upregulated DEGs which were the KO terms related to secondary metabolite biosynthesis in the BT stage transcriptomes. **(C)** Hierarchical cluster analysis of the downregulated DEGs which were KO terms related to secondary metabolite biosynthesis in the BT stage transcriptomes. The red color denotes the highly expressed up-regulated DEGs, and the green color denotes down-regulated DEGs with lower expression levels. The gradation from red to green represents the transition from large to small values of a FPKM normalized log_2_ transformed counts. The sample names are indicated below each column. DEGs are defined by different colors, with the normalized expression levels employing a color gradient from low (green) to high (red).

In the BT stage, genes that were upregulated in response to the knockdown of *NtERF189/199* were significantly enriched in several GO terms, including glutathione metabolic process (GO:0006749), response to temperature stimulus (GO:0009266), diterpenoid biosynthetic process (GO:0016102), and glycogen metabolic process (GO:0005977). For example, the GO term response to temperature stimulus encompassed genes from the heat shock protein 20 family, which are known for their role in responding to a variety of abiotic and biotic stresses. On the contrary, downregulated genes in the BT stage were primarily associated with GO terms related to calcium ion binding (GO:0005509), xyloglucan metabolic process (GO:0010411), ubiquitin-protein transferase activity (GO:0004842), protein serine/threonine kinase activity (GO:0004674), and sequence-specific DNA binding (GO:0043565) (**Figure 4**). In the initial plant immunity steps, specific cytosolic Ca^2+^ rises in the cytosol and in organelles, then these Ca^2+^ specifically binds to a plethora of sensors including calmodulin (CaM), CaM-like proteins (CML), calcium-dependent protein kinases (CDPK), which, in turn, activate target proteins either through direct binding or phosphorylation (Aldon et al., 2018). Additionally, the GO terms sequence-specific DNA binding includes different stress-induced TF family genes, such as *ERF*, *WRKY*, jasmonate ZIM domain (*JAZ*) proteins and *GRAS*. These genes were mostly downregulated by knocking down of *NtERF189/199* in the BT transcriptomes.

In the T0.5 stage, there were more upregulated DEGs compared to downregulated genes (353 upregulated and 63 downregulated). These upregulated DEGs were enriched in GO terms associated with catalytic activity (GO:0003824), protein binding (GO:0005515), and ion binding (GO:0043167). These terms included genes belonging to ribosomal proteins, chlorophyll A-B binding proteins, cytochrome P450, photosystem-related proteins, and heat shock proteins (HSPs) (**Supplementary Table 6**). Notably, the presence of a significant number of DEGs related to HSPs in both BT and T0.5 transcriptomes suggests their essential role in response to nicotine deficiency. HSPs play vital roles in accumulating resistance proteins and coordinating defense signaling cascades in plants (Park and Seo, 2015). Additionally, HSPs act as crucial components in both prokaryotic and eukaryotic organisms by preventing protein aggregation during abiotic stresses (Muthusamy et al., 2017; Hu et al., 2022). HSPs can be categorized into different groups based on their molecular weights, including large HSPs, HSP90, HSP70, HSP60, HSP20, and small HSPs (Hu et al., 2022). These chaperone proteins help maintain cellular proteostasis, facilitate protein synthesis, folding, degradation, and signal transduction. Expression of HSP20s in plants is known to be induced by various stressors, including heat, drought, osmotic stress, phytohormones, and oxidative stresses (Guo et al., 2020). To be specific, a number of *HSP* genes belonging to *HSP20*, *HSP70*, and *HSP90* classes were significantly upregulated in response to topping in low nicotine tobacco. The increased expression of HSP genes in low-nicotine tobacco may represent a strategy to enhance overall stress tolerance and compensate for the loss of pyridine alkaloids in tobacco. Furthermore, the upregulation of genes related to ribosomes in response to nicotine deficiency suggests their role in translational regulation during the protein synthesis process. In fact, ribosomes play a housekeeping role in translational regulation during protein synthesis process (Horiguchi et al., 2012). The 30S ribosomal subunit works with the 50S subunit to ensure genetic message translation accuracy and move the tRNAs to associated mRNA for translocation (Carter et al., 2000). Accumulating evidence suggested that loss of ribosomal proteins result in a reduced growth rate and developmental defects in plants. For instance, *ribosomal protein L27* (*RPL27*), *RPL11*, and *ribosomal protein S1*(*RPS1*) are known regulatory genes for growth and patterning, photosynthesis, heat stress-responsive gene expression in Arabidopsis (Pesaresi et al., 2001; Szakonyi and Byrne, 2011; Yu et al., 2012b). The upregulation of *Nicotiana* homologous genes related to ribosomal proteins in low-nicotine tobacco further supports their importance in plant defesive responses. Moreover, the genes related to carbohydrate metabolism (GO:0005975), including galactinol synthase (*GolS*), glucan endo-1,3-beta-glucosidase, and inositol-3-phosphate synthase, which functions in responding to abiotic and biotic stresses, fungal pathogen resistance, and salt tolerance, are upregulated in low nicotine condition (**Supplementary Table 6**).

**Figure 5:**
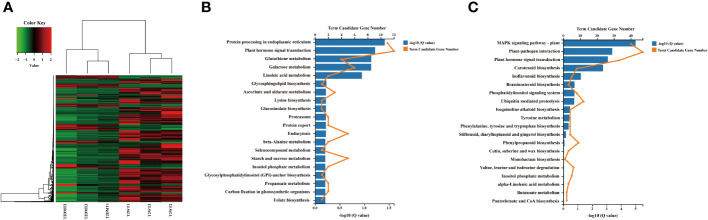
Hierarchical cluster analysis and the KEGG enrichment of the DEGs identified in the T2 stage transcriptomes. **(A)** Heatmap analysis of DEGs based on the KO terms in secondary metabolite biosynthesis in the T2 transcriptomes. The red color denotes the highly expressed up-regulated DEGs, and the green color denotes down-regulated DEGs with lower expression levels. The gradation from red to green represents the transition from large to small values of a FPKM normalized log_2_ transformed counts. The sample names are indicated below each column. DEGs are defined by different colors, with the normalized expression levels employing a color gradient from low (green) to high (red). **(B)** KEGG enrichment analysis of the upregulated DEGs identified in the T2 transcriptomes. **(C)** KEGG enrichment analysis of the down-regulated DEGs identified in the T2 transcriptomes. The blue column represents the degree of significance of the enrichment of DEGs in a pathway is indicated by -log_10_ (Q value). The x-axis represents the number of DEGs annotated to a particular pathway.

In the T2 stage, we observed that most of the DEGs were downregulated (175 upregulated and 574 downregulated) when compared to wild-type plants. The downregulated genes in this stage were associated with GO terms related to metabolic processes (GO:0008152) and biological regulation (GO:0065007). They also had molecular functions related to calcium ion binding (GO:0005509), heterocyclic compound binding (GO:1901363), nitrogen compound transport (GO:0071705), DNA-binding transcription factor activity (GO:0003700), carotenoid dioxygenase activity (GO:0010436), and ubiquitin-protein transferase activity (GO:0004842) (**Supplementary Table 6**). These downregulated genes belonged to various gene families, including calmodulin-binding proteins, cytochrome P450 enzymes, exocyst complex component proteins, plant transcription factor genes (*ERF*, *MYB*, Zinc-finger, *NAC*, *GRAS*, and *bHLH*), epoxycarotenoid dioxygenase genes, and U-box domain-containing proteins. Notably, the plant U-box proteins (PUBs) were among the downregulated genes. *PUBs* are closely associated with protein kinases involved in stress signaling and development and paired with E2-ubiquitin-conjugating enzymes to participate in signal hubs and mark substrates for degradation (Azevedo et al., 2001; Trenner et al., 2022). The *OsPUB* protein *spotted leaf11* (*spl11*) mutant showed enhanced rice blast and bacterial blight resistance (Zeng et al., 2004). Expression of *PUB22*, *PUB23*, and *PUB24* was induced by pathogen infections and *pub22/pub23/pub24* triple mutant displayed enhanced activation of PAMP-triggered immunity as well as increased resistance against pathogens in *Arabidopsis* (Trujillo et al., 2008). PUB22 and PUB23 also showed to interact with ABA receptor pyrabactin resistance-like 9 (PYL9) to facilitate its own degradation and increase plant drought tolerance in *Arabidopsis* (Zhao et al., 2017). In addition to PUBs, the downregulated DEGs included members of the cytochrome P450 family, which are involved in various aspects of secondary metabolite biosynthesis and phytohormone signaling (GO:0009695) (Pandian et al., 2020). For instance, the expression of CYP450 proteins such as *TRANSPARENT TESTA 7* (*TT7*) and *allene oxide synthase* (*AOS*) was suppressed. These genes are well-known structural genes involved in flavonoid biosynthesis and alpha-linolenic acid metabolism in Arabidopsis. Furthermore, this study also suggests that nicotine reduction in tobacco has a significant impact on the expression of various receptor serine/threonine kinase (RSTK) genes, which are a subclass of receptor-like kinase (RLK) proteins (**Figure 5**; **Supplementary Table 6**). Plant receptor-like kinase (RLK) proteins interact with diverse group of proteins to determine combinatorial variations in signal response specificity in the process of pathogen recognition, plant defense mechanism activation (Afzal et al., 2008). Receptor protein kinase can be divided into three subclasses according to different substrate specificities among the kinase domains, including receptor tyrosine kinase (RTK), receptor serine/threonine kinase (RSTK), and receptor histidine kinase (RHK), respectively (Becraft, 2002). RSTK genes, known as the receptor-like kinase, are involved in cell growth and development (Li and Chory, 1997; Jinn et al., 2000) as well as plant-pathogen interaction and defense responses (Song et al., 1995; Gómez-Gómez and Boller, 2000) in plants. For instance, three *CLAVATA*-like genes *CLV1/CLV2/CLV3* are involved in meristem-size homeostasis and loss of function of each of them result in a shoot meristem enlargement phenotype in Arabidopsis (Clark et al., 1997). In addition, the receptor kinase FLS2 interacts with bacterial elicitor to activate its kinase domain which in turns to initiate downstream signaling for defense response in Arabidopsis (Haffani et al., 2004; Chinchilla et al., 2006). These results indicates that *NtERF189/199* appear to play central roles in play central roles in enhancing plant defense signaling cascades and, in turn, activating the biosynthesis of specialized metabolites in tobacco.

Collectively, these findings suggest that the expression intensity of a wide range of genes associated with various biological processes, such as biotic and abiotic stress responses, secondary metabolite biosynthetic pathways, and transcriptional regulations, were significantly decreased in response to knocking down *NtERF189/199* when compared to the wild-type control. This reduction in gene expression in these crucial pathways reflects the broader impact of nicotine reduction on the plant’s overall physiology and defense mechanisms. In contrast, genes involved in heat shock responses, carbohydrate metabolism, ribosomal proteins, and plant U-box proteins were upregulated and are likely compensatory mechanisms that help offset the negative effects of nicotine synthesis defects in low nicotine tobacco. The study’s results provide a global perspective on why nicotine reduction in tobacco can render *Nicotiana* species more vulnerable to a wide range of biotic and abiotic stresses, more attractive targets for herbivore attacks compared to wild-type plants. A complete list of GO terms associated with three different growing stages were summarized in **Supplementary Table 6**.

### KEGG pathway enrichment and functional classification

**Figure 6:**
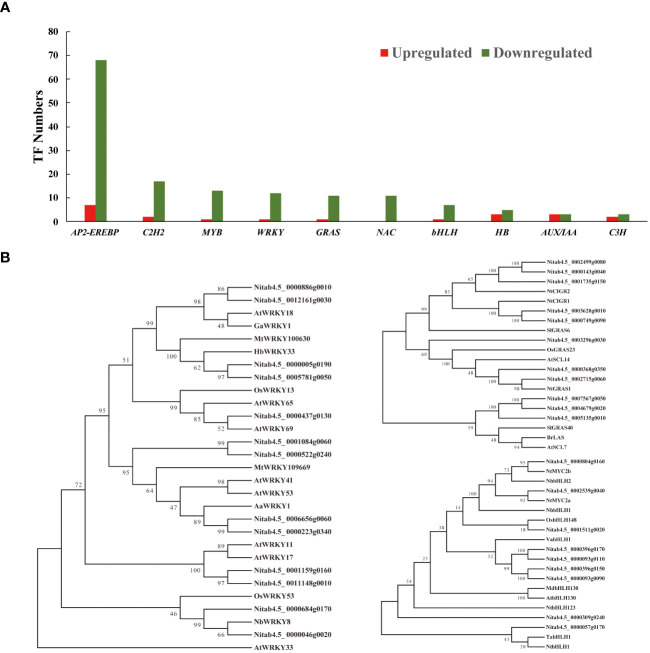
The transcription factor statistics and phylogenetic analysis. **(A)** The statistical analysis of the transcriptional regulators identified in all transcriptomes. **(B)** Amino acid sequences of three TF family genes (WRKY, GRAS, and bHLH) were selected to construct individual phylogenetic trees using MEGA-X software with the neighbor-joining method (with 2000 replications). Bootstrap values are indicated at branch nodes.

KEGG pathway enrichment analysis was performed to assign biological functions to these DEGs within known biological pathways in response to alkaloid reduction in tobacco, with particular emphasis on the T2 stage. This stage is of particular interest due to its potential implications for the differential synthesis of metabolites in wild-type and ultra-low nicotine tobacco plants. The enriched specialized pathways were separately categorized into two groups based on their expression status (|log2-fold change| ≥1) to visualize affected pathways and predict differentially accumulated metabolites (**Figure 6**). In the nicotine-deficient condition, most of upregulated DEGs were significantly enriched in the classes of galactose metabolism (sly04075, 24.00%), amino sugar and nucleotide sugar metabolism (sly00940, 19.00%), carbon metabolism (16.00%), and starch and sucrose metabolism (sly00592, 10.00%). In detail, genes encoding enzymes in raffinose family oligosaccharides, e.g. alkaline alpha galactosidase (Nitab4.5_0002649g0010 and Nitab4.5_0009705g0010) and galactinol synthase (Nitab4.5_0001013g0090), were upregulated in response to the lack of alkaloid accumulation (**Figure 6A**; **Supplementary Table 7**). Additionally, genes related to glycoside hydrolase (glucan endo-1,3-beta-glucosidase 5), glycosyl hydrolase (1,4-alpha-glucan branching enzyme II) and glycolysis (triosephosphate isomerase and glyceraldehyde-3-phosphare dehydrogenase) were also significantly induced (**Figure 6A**; **Supplementary Table 7**). In addition, alkaloid reduction in tobacco also triggered the activation of pathways leading to the biosynthesis of other specialized metabolites, such as betalains (Nitab4.5_0002352g0120), indole alkaloids (Nitab4.5_0010352g0040 and Nitab4.5_0001048g0050), isoquinoline alkaloids (Nitab4.5_0010352g0010), phenylpropanoids (Nitab4.5_0016891g0010), and sesquiterpenoids and triterpenoids (Nitab4.5_0000040g0410) (**Figure 6B**; **Supplementary Table 7**). Conversely, genes related to certain biosynthetic pathways were downregulated in the ultra-low nicotine condition, such as the biosynthesis of amino acids (valine, leucine, isoleucine, cysteine, methionine, and arginine), ascorbate and aldarate metabolism, and carotenoid biosynthesis. For example, genes related to amino acid biosynthesis such as arginine decarboxylase (Nitab4.5_0005266g0020), aminotransferases (Nitab4.5_0002231g0020), tryptophan synthase (Nitab4.5_0002155g0050) and prephenate dehydratase (Nitab4.5_0008983g0010) were downregulated (**Figure 6B**; **Supplementary Table 7**). Also, KEGG enrichment analyses indicated the DEGs were significantly downregulated in the various pathways like carotenoid biosynthesis (Nitab4.5_0001070g0130 and Nitab4.5_0000192g0200), biosynthesis of cofactors (Nitab4.5_0010352g0010), brassinosteroid biosynthesis (Nitab4.5_0000364g0040 and Nitab4.5_0002898g0020), and pentose and glucuronate interconversions (Nitab4.5_0000741g0040 and Nitab4.5_0005235g0040) (**Figure 6B**; **Supplementary Table 7**). Taken together, these results indicate that the knockdown of *NtERF189/199*, two dominant activators for nicotine synthesis, has a substantial impact on the metabolic reprogramming and metabolite accumulation. The plant appears to compensate for nicotine deficiency by modulating the biosynthesis of other specialized metabolites.

### Nicotine reduction suppresses TF expression intensity involved in disease resistances, environmental stress tolerances and secondary metabolism

To investigate the wide-ranging impact of nicotine synthesis deficiency on transcriptional regulators, we conducted a systematic analysis across our transcriptomes. Our analysis revealed key TFs representing diverse families, including *AP2-EREBP*, *C2H2*, *MYB*, *WRKY*, *GRAS*, *NAC*, and *bHLH*, which play pivotal roles in modulating both defense responses and secondary metabolism as either positive or negative regulators (**Figure 6A**). Among these TF families, the *AP2-EREBP* family is most noticeable, encompassing a substantial number of *ERFs*. Previous studies have identified at least 239 *AP2/ERF* TFs in tobacco genome can be classified into ten subgroups, from group I to X (Rushton et al., 2008). *AP2/ERF* TFs comprise a diverse and influential group of factors that play essential roles in regulating various biological and physiological processes in plants (Licausi et al., 2013). These processes include plant morphogenesis, phytohormone signaling, responsiveness to environmental stimuli, and the biosynthesis of specialized metabolites (Feng et al., 2020). Our analysis identified a total of 75 *AP2/ERF* TFs (7 upregulated and 68 downregulated) displaying differential expression patterns, which could be further categorized into groups III, VII, IX, and X, based on phylogenetic analysis. Notably, several *ERF* TFs within this family have been recognized for their involvement in regulating a wide array of responses to biotic and abiotic stresses in tobacco. This group includes previously documented members such as *NtERF1*, *NtERF5*, *NtERF172*, *ACRE111a*/*111b*, *ACRE1*, and *EREBP2*/*3/5/6* (**Supplementary Table 5**). EREBP proteins, functioning as activators or repressors, are known to specifically bind to the ethylene-responsive element (GCC-box) and regulate ethylene-responsive gene transcription (Ohme-Takagi and Shinshi, 1995; Ohta et al., 2001). For instance, NtERF5 has demonstrated enhanced resistance to tobacco mosaic virus (TMV), accompanied by reduced hypersensitive-response lesions, suggesting a role in controlling viral propagation (Fischer and Dröge-Laser, 2004). Additionally, NtERF172 has been identified as a positive regulator of tobacco drought resistance, achieved by direct interaction with the catalase *NtCAT* gene promoter to enhance CAT-mediated H_2_O_2_ homeostasis in tobacco (Zhao et al., 2020). Furthermore, the Tobacco Avr9/Cf-9 rapidly elicited (*ACRE*) genes, induced by elicitors and wounding, encode putative signaling components that initiate the defense response (Rowland et al., 2005).

Furthermore, the impact of alkaloid reduction extended to the downregulation of *WRKY* TFs, which are extensively recognized for their pivotal roles in plant innate immunity, serving as responsive genes to pathogen infections, elicitors, and phytohormone inductions. *WRKY* TFs contribute significantly to regulating both biotic and abiotic stress tolerance and play a substantial role in specialized metabolism in plants (Schluttenhofer and Yuan, 2015). Our transcriptomic analysis revealed a considerable decrease in the expression levels of these differentially expressed *WRKY* TFs in comparison to the control. Phylogenetic analysis suggested that these WRKY protein orthologs have functions in defense responses and secondary metabolite biosynthesis in various species, including Arabidopsis, wheat, *Oryza sativa*, and *N. benthamiana*. Notably, we identified two orthologous genes (Nitab4.5_0000046g0020 and Nitab4.5_0000684g0170) clustered with NbWRKY8 (**Figure 6B**). Previously, phosphorylation of *NbWRKY8* induce the expression of defense-related genes, specifically *3-hydroxy-3-methylglutaryl CoA reductase 2* and *NADP-malic enzyme* expression. VIGS-based silencing of *WRKY8* leads to increased disease susceptibility to *Phytophthora* and *Colletotrichum* pathogens (Ishihama et al., 2011). Furthermore, AaWRKY1, which cladding with two tobacco WRKY genes (Nitab4.5_0000665g0060 and Nitab4.5_0000223g0340), serves as a positive regulator of jasmonic acid (JA)-mediated signaling. It enhances artemisinin biosynthesis by trans-activating the expression of the *amorpha-4,11-diene synthase* gene in *Artemisia annua* (Ma et al., 2009). Additionally, overexpression of *OsWRKY13* has been demonstrated to activate the expression of salicylic acid (SA) synthetic genes while simultaneously repressing JA signaling-responsive genes, thus increasing resistance to bacterial blight and fungal blast in rice (Qiu et al., 2007). Previous studies have shown that HbWRKY3, which is JA-inducible, plays a significant role in regulating rubber biosynthesis in *Hevea brasiliensis* (Li et al., 2014). Similarly, *Medicago truncatula* WRKY100630 is known to activate the biosynthesis of phenolic compounds and lignin accumulation in transgenic tobacco (Naoumkina et al., 2008). Notably, two NtWRKY proteins (Nitab4.5_0000886g0010 and Nitab4.5_0012161g0030) are affiliated with these two WRKY TFs, further suggesting their role as positive regulators in JA signaling and lignin biosynthesis (**Figure 6B**).

Significantly, we also observed the downregulation of several GRAS family TFs in the transcriptomes of low-nicotine tobacco (**Figure 6B**). GRAS family genes are known for their multifaceted roles in plant growth, development, and stress tolerance (Waseem et al., 2022). Notably, we identified five tobacco GRAS TFs (Nitab4.5_0002499g0080, Nitab4.5_0000143g0040, Nitab4.5_0001735g0150, Nitab4.5_0003620g0010, and Nitab4.5_0000749g0090) that are phylogenetically related to their orthologous genes in rice and tomato, which are implicated in responses to both biotic and abiotic stress. In rice, CIGR1 and CIGR2, members of the GRAS gene family, are significantly induced by exogenous gibberellins in a dose-dependent manner, and their expression is regulated by phosphorylation cascades (Day et al., 2004). Specifically, GIGR2 acts as a transcriptional activator of the B-type heat shock protein *OsHsf23*, enhancing disease resistance to rice blast fungus infection (Tanabe et al., 2016). Similarly, in tomato, the transcripts of SlGRAS6 and its orthologue preferentially accumulate in response to avirulent bacteria and fungal elicitors. Suppression of SlGRAS6 through VIGS-mediated gene silencing results in impaired drought tolerance (Mayrose et al., 2006). The downregulation of three tobacco GRAS family genes (Nitab4.5_0003296g0030, Nitab4.5_0000368g0350, and Nitab4.5_0002715g0060) in conjunction with the low-nicotine phenotype aligns with previous findings indicating that low nicotine tobacco exhibits reduced disease tolerance and stress resistance in tobacco (Steppuhn et al., 2004). Furthermore, overexpression of stress-induced *OsGRAS23* has been demonstrated to confer improved drought resistance and oxidative stress tolerance in rice plants (Xu et al., 2015). Tobacco NtGRAS1 is another example, as it is strongly induced by various environmental stresses and correlates with increased intracellular reactive oxygen species (ROS) levels (Czikkel and Maxwell, 2007). Similarly, GRAS family TFs, such as SlGRAS40, BrLAS, and AtSCL7, have been identified as positive regulators of drought and salinity tolerance in tomato, Brassica, and Arabidopsis (Ma et al., 2010; Liu et al., 2017; Li et al., 2018). The expression of their tobacco orthologues (Nitab4.5_0007567g0050, Nitab4.5_0004679g0020, and Nitab4.5_0005135g0010) in our transcriptome was also notably decreased (**Figure 6B**).

The bHLH TF family is known for its diverse and vital roles in various biological processes within plants. Notably, a bHLH TF, NtMYC2a, has been identified as a master regulator in the JA (jasmonic acid) signaling transduction cascade, functioning upstream to regulate nicotine synthesis through *ERF* clusters (Shoji et al., 2010; Sui et al., 2020; Hou et al., 2023). In our study, the homologs of NtMYC2a/b (Nitab4.5_0002539g0040 and Nitab4.5_0000804g0160) were also found to be downregulated in low-nicotine tobacco leaves upon the knockdown of *NtERF189* and *NtERF199*. In wheat, the transcript level of the *TabHLH1* gene is induced by different osmotic stresses, phosphate, and nitrogen deprivation. Overexpressing *TabHLH1* in transgenic tobacco results in enhanced drought resistance and salinity tolerance, presumably through its modulation of the ABA signaling pathway, nutrient transporter genes, and ROS homeostasis (Yang et al., 2016). Another example is NtbHLH1, which functions as a transcription activator and responds to iron deficiency by activating the expression of iron deficiency-related genes (Li et al., 2020). In our study, the expression of orthologues of *TabHLH1* and *NtbHLH1* in tobacco (Nitab4.5_0000057g0170) was also found to be repressed along with the decline in nicotine levels in tobacco leaves. Furthermore, the stress-inducible gene *OsbHLH148*, a homolog of the tobacco *bHLH* TF (Nitab4.5_0001511g0020), has been demonstrated to respond to environmental stresses and phytochrome inductions. It interacts with the OsJAZ1 protein to initiate jasmonate-regulated gene expression, contributing to drought tolerance (Seo et al., 2011). Collectively, these findings suggest that *NtERF189* and *NtERF199* not only play a role in nicotine biosynthesis and accumulation for insect resistance but also interconnecting with the regulatory mechanisms of *ERF*, *WRKY*, *GRAS*, and *bHLH* TFs. These TFs exhibit functional versatility, responding to a wide range of biotic and abiotic stresses in plants.

### Transcriptomic expression level validation and biotic stress tolerance assays

**Figure 7:**
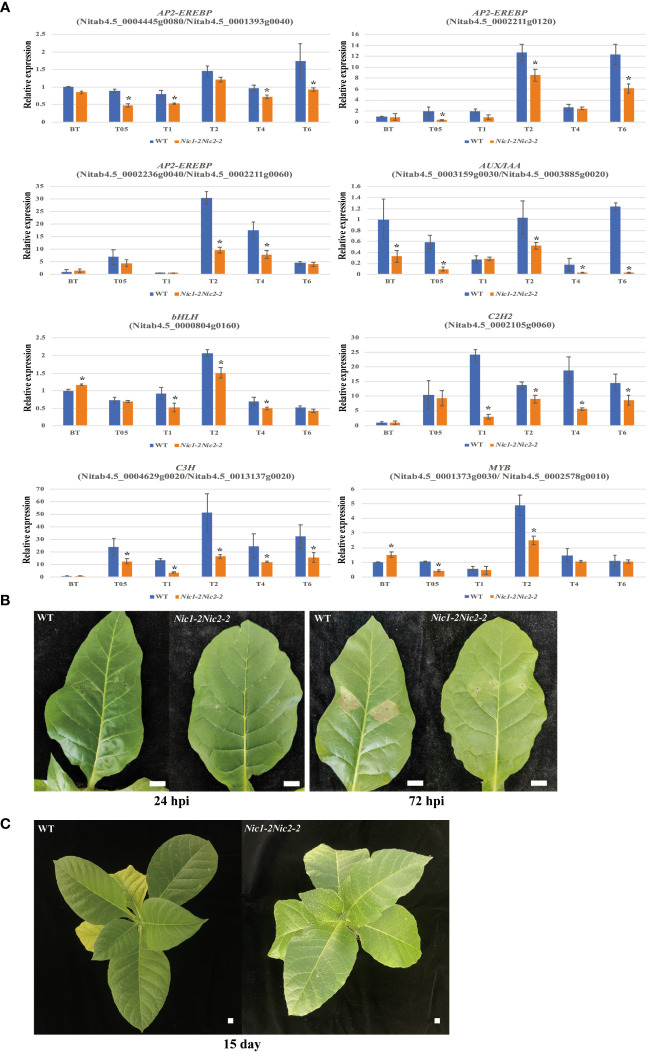
Verification of the expression level of stress-related genes and biotic stress tolerances in low nicotine tobacco plants. **(A)** qRT-PCR validation of the downregulated DEGs related to transcriptional regulation in low nicotine plants. The relative expression levels of 8 candidate genes and their homologous genes (*AP2-EREBP*, *AUX/IAA*, *bHLH*, *C2H2*, *C3H* and *MYB*) in the leaves of the *Nic1-2Nic2-2* lines. The actin gene (accession No.: X63603) was used as an internal control. Data represent means ± standard deviation (SD) of three replicates. *P<0.05(n=3), by Student’s t-test. **(B)** Transient expression of TMV-Cg CP in wild type (WT) and low nicotine plant (*Nic1-2 Nic2-2*). The TMV-Cg CP was transiently expressed in *N. tabacum* leaves using agroinfiltration. As the *N. tabacum* contains *N’* gene, which is resistance to TMV-Cg, the transient expression of TMV-Cg CP could induce hypersensitive response (HR) symptoms in infiltrated leaves. At 24 hours post infiltration (hpi), the WT leaves exhibited initial HR symptoms, while the *Nic1-2Nic2-2* plants has no HR symptoms. At 72 hours post infiltration (hpi), the WT leaves exhibited solid HR symptoms, while the *Nic1-2Nic2-2* plants has only mild HR symptoms. **(C)** The feeding preference of tobacco aphid to WT and *Nic1-2Nic2-2* plants. The same amount of aphid larva was placed on *N. tabacum* seedling leaves. After fed for 15 days, the nicotine-deficient seedlings (*Nic1-2Nic2-2*) are more susceptible to *Myzus persicae* and attract significantly higher number of aphid than did WT plants. Bar = 1 cm.

Based on the KEGG results obtained by RNA-seq analysis, we validated the expression levels of genes that belong to different TF families by using qRT-PCR analysis. As expected, the expression patterns of these selected TF genes analyzed were consistent with the transcriptomic data (**Figure 7A**). For example, expression of these *ERF* genes and their homologs (Nitab4.5_0004445g0080 and Nitab4.5_0001393g0040; Nitab4.5_0002236g0040 and Nitab4.5_0002211g0060; Nitab4.5_0002211g0120) was significantly downregulated at different growing stages in the *Nic1-2Nic2-2* transgenic lines, compared with the WT control (**Figure 7**). In addition, we also measured the expression levels of one *bHLH* gene (Nitab4.5_0000804g0160) and one *C2H2* gene (Nitab4.5_0002105g0060). Similarly, expression of these two transcripts was also significantly reduced in several growing stages by the loss-of-function *NtERF189* and *NtERF199* (**Figure 7**). Moreover, we also checked expression of other TF family genes, including two *C3H* homologous genes (Nitab4.5_00044629g0020 and Nitab4.5_0013137g0020), *AUX/IAA* homologous genes (Nitab4.5_0003159g0030 and Nitab4.5_0003885g0020), and *MYB* homologous genes (Nitab4.5_0001373g0030/Nitab4.5_0002578g0010), and these genes were also significantly decreased in the loss-of-nicotine transcriptomes. Furthermore, transient expression of TMV-Cg CP in WT and *Nic1-2 Nic2-2* were carried out. The TMV-Cg CP was transiently expressed in *N. tabacum* leaves using agroinfiltration. As the *N. tabacum* contains *N’* gene, which is resistance to TMV-Cg, the transient expression of TMV-Cg CP could induce hypersensitive response (HR) symptoms in infiltrated leaves. As illustrated, at 24 hours post infiltration (hpi), the WT leaves exhibited initial HR symptoms, while the *Nic1-2 Nic2-2* plants has no HR symptoms. At 72 hpi, the WT leaves exhibited solid HR symptoms, while the *Nic1-2 Nic2-2* plants has only mild HR symptoms (**Figure 7B**). The HR implies that the *N. tabacum* plants are resistant to TMV-Cg, whereas the severity of HR indicates the resistance level of WT and *Nic1-2 Nic2-2* plants. The postponed and milder HR symptom in *Nic1-2 Nic2-2* plants indicated that the resistance level was down-regulated in these nicotine deficient plants compared with WT. Similarly, the aphid feeding preference assay demonstrated that the low nicotine seedlings (*Nic1-2Nic2-2*) are more susceptible to *Myzus persicae* and attract significantly higher number of aphid than did WT plants after feeding with aphid larvae for 15 days (**Figure 7C**). Taken together, these results confirmed the reliability of our transcriptome data and demonstrate that nicotine resistance trait is tightly linked to control the expression levels of various abiotic and biotic stresses related and quantitative resistance related genes *in vivo* to increase its own environmental fitness.

## Discussion

Alkaloids represent a diverse group of nitrogen-containing compounds produced in various plant species, and they have been attributed multiple functions, including serving as nitrogen reserves, aiding in detoxification processes, regulating plant growth, and acting as a defense mechanism against insects and predatory herbivores (Bush et al., 1993; Ali et al., 2019). Nicotine, a well-documented defensive alkaloid in *Nicotiana* species, is also employed as an insecticide in agricultural applications (Shepard, 1951; Schmeltz, 1971; Baldwin, 1996; Baldwin et al., 1997; Baldwin, 1998, Baldwin, 2001; Voelckel et al., 2001). Herbivore attack triggers a *de novo* nicotine biosynthesis through induce jasmonic acid signaling transduction cascade achieved by trans-activating multiple transcriptional regulators (Kazan and Manners, 2008, Kazan and Manners, 2013; Shoji and Hashimoto, 2011a; Shoji and Hashimoto, 2011b; Shoji et al., 2008; Shoji and Hashimoto, 2013, Shoji and Hashimoto, 2015, Shoji et al., 2010; Zhang et al., 2012; Sears et al., 2014; Sui et al., 2019; Sui et al., 2020; Qin et al., 2021; Godbole et al., 2022).

Historically, the pursuit of breeding low-nicotine tobacco dates back to the 1940s with the primary objective was to mitigate the throat irritation caused by nicotine during cigar inhalation (Valleau, 1949). The low alkaloid trait from Cuba cigar varieties were then incorporated into Burley and flue-cured tobaccos using traditional breeding methods (Legg et al., 1969; Chaplin, 1975). Genetic studies indicate that the level of total alkaloids in tobacco is primarily regulated by two independent loci, denoted as A and B (also referred to as *NIC1* and *NIC2*, respectively) (Legg et al., 1969; Legg and Collins, 1971). Consequently, different nearly isogenic lines (NILs) have been developed, carrying recessive alleles at the *Nic1* and/or *Nic2* loci, resulting in four distinct levels of alkaloids in tobacco, namely high alkaloid, high-intermediate, low-intermediate, and low alkaloid (Chaplin, 1975; Chaplin and Burk, 1984, and Chaplin, 1986; Legg et al., 1970; Nielsen et al., 1988). However, the allelic variability in nicotine content was also found to have pleiotropic effect on tobacco agronomic traits, such as yield, leaf maturation, reducing sugar, polyamines levels, mesophyll cells and chlorophyll contents (Chaplin and Weeks, 1976; Nölke et al., 2018).

Recent advancements in tobacco genomics have facilitated a more comprehensive understanding of the physical locations and genetic compositions of the *NIC1* and *NIC2* loci within the tobacco genome (Shoji et al., 2010; Kajikawa et al., 2017; Sui et al., 2019; Sui et al., 2020). It has been established those recessive alleles of *Nic1* and *Nic2* in natural nicotine mutants result from chromosomal deletions (Shoji et al., 2010; Kajikawa et al., 2017) and chromosomal deletion-induced structural variations (Sui et al., 2020; Shoji et al., 2010). However, it’s important to note that allelic variations arising from naturally occurring mutations in these nearly isogenic lines (NILs), which were generated through repeated backcrossing, can introduce confounding variations. These variations may encompass not only the regulatory genes themselves but also genomic structural changes that could potentially be linked to both agronomic traits and the physiological alterations related to nicotine. A supporting observation in this scenario is that *Nic1-2* and *Nic2-2* double mutants did not exhibit any discernible abnormalities in terms of growth and development during laboratory trials (Hayashi et al., 2020).

For precise estimation of specific defense traits in plants, it is ideal to work with plant mutants that differ only in a single gene controlling the expression of the target trait while all other characteristics are identical (Bergelson and Purrington, 1996; Steppuhn et al., 2004). To reduce nicotine and other alkaloid levels, many attempts have been employed, including the reduction of expression levels or specific mutations of nicotine synthesis structural genes and their transcriptional regulators (such as *NtQPT*, *NtPMTs*, *NtBBLs*, *NtA622s*, *NtMPO*) using either RNA interference or CRISPR/Cas9-based editing technologies (Conkling et al., 2001; Chintapakorn and Hamill, 2003; Steppuhn et al., 2004; Wang et al., 2009; Lewis et al., 2015; Lewis, 2019; Hayashi et al., 2020; Sui et al., 2020; Burner et al., 2022). For example, silencing the expression of *PMT* in *Nicotiana attenuata* has shown that transgenic plants become more attractive to herbivore attacks by nicotine-adapted herbivores compared to wild-type plants (Steppuhn et al., 2004). In line with this rationale, the creation of nicotine mutants by simultaneously loss-of-function of *NtERF199* and *NtERF189*, resulting in reduced biotic and abiotic stress tolerance, precisely meets the requirements for studying nicotine’s role in plant defense resistance in tobacco (**Figure 7**). Therefore, it is significance to provide a comprehensive transcriptomic overview of the transcriptional and metabolic differences between *Nic1-2Nic2-2* mutants and wild-type plants.

In this study, we conducted a comparative transcriptomic analysis to provide additional evidence supporting the notion that the nicotine synthesis regulon closely coordinates the intensity of gene expression related to various aspects of stimulus perceptions, signal transduction, and defensive response in plant innate immunity system. The individual sequencing library generated more than 56 M reads with more than 90% mapping rates on the reference genome suggests the high quality and sequencing depth of the library (**Supplementary Tables 1**, **2**). The differential gene expression patterns in each of the investigated stages, ranging from 68 to 880, exhibited distinct transcriptional patterns between the *Nic1-2Nic2-2* mutants and the wild-type plants, indicating that nicotine deficiency significantly altered the gene expression and metabolic consequence in tobacco. Notably, three stages (BT, T05, and T2) exhibited a higher number of DEGs in the *Nic1-2Nic2-2* mutants compared to the wild-type control (**Figure 1**).

Nicotine, a well-known pyridine alkaloid, serves as a prominent plant resistance trait in *Nicotiana* species. Loss of function in nicotine production renders *N. attenuata* more attractive to tobacco-chewing insects (Steppuhn et al., 2004). Our Gene Ontology (GO) enrichment analysis has shown that the biological processes associated with these GO terms related to cellular processes (GO:0009987), metabolic processes (GO:0008152), responses to stimuli (GO:0050896), biological regulation (GO:0065007), and signaling (GO:0023052) are significantly altered (**Figure 3**). The downregulation of DEGs associated with calcium ion binding (GO:0005509), protein serine/threonine kinase activity (GO:0004674), and ubiquitin-protein transferase activity (GO:0004842) suggests that plant pathogen perception within the innate immune system, to some extent, is compromised in low-nicotine plants. In signal transduction process, the expression levels of DEGs related to the MAP kinase cascade and sequence-specific DNA binding (GO:0043565), which includes members of the *ERF*, *WRKY*, *bHLH*, and *GRAS* TF families, are significantly reduced. This reduction implies a limited capacity for downstream signal transduction to induce sufficient critical defense and metabolic processes, such as the production of reactive oxygen species and the biosynthesis of various specialized metabolites. Indeed, several GO terms related to metabolic processes (GO:0008152) also exhibit significant weakening. These terms include xyloglucan metabolic process (GO:0010411), heterocyclic compound binding (GO:1901363), nitrogen compound transport (GO:0071705), and carotenoid dioxygenase activity (GO:0010436) (**Supplementary Table 6**). Different from WT plants, low-nicotine tobacco appears to reconfigure its innate immune system through the activation of other specific GO processes. These include temperature stimulus (GO:0009266), glycogen metabolic processes (GO:0005977), catalytic activity (GO:0003824), carbohydrate metabolism (GO:0005975), and diterpenoid biosynthetic processes (GO:0016102), evident by the upregulation of key genes belonging to various families, such as heat shock proteins, ribosomal proteins, galactinol synthases, glucan endo-1,3-beta-glucosidases, and alpha-farnesene synthases. Furthermore, this unique downstream signaling cascade under nicotine-deficient conditions has the potential to uncover a new subset of TF family genes with functions related to enhancing plant-pathogen resistance. Typically, the contributions of these genes are probably overlooked in wild-type tobacco (**Supplementary Table 6**).

**Figure 8:**
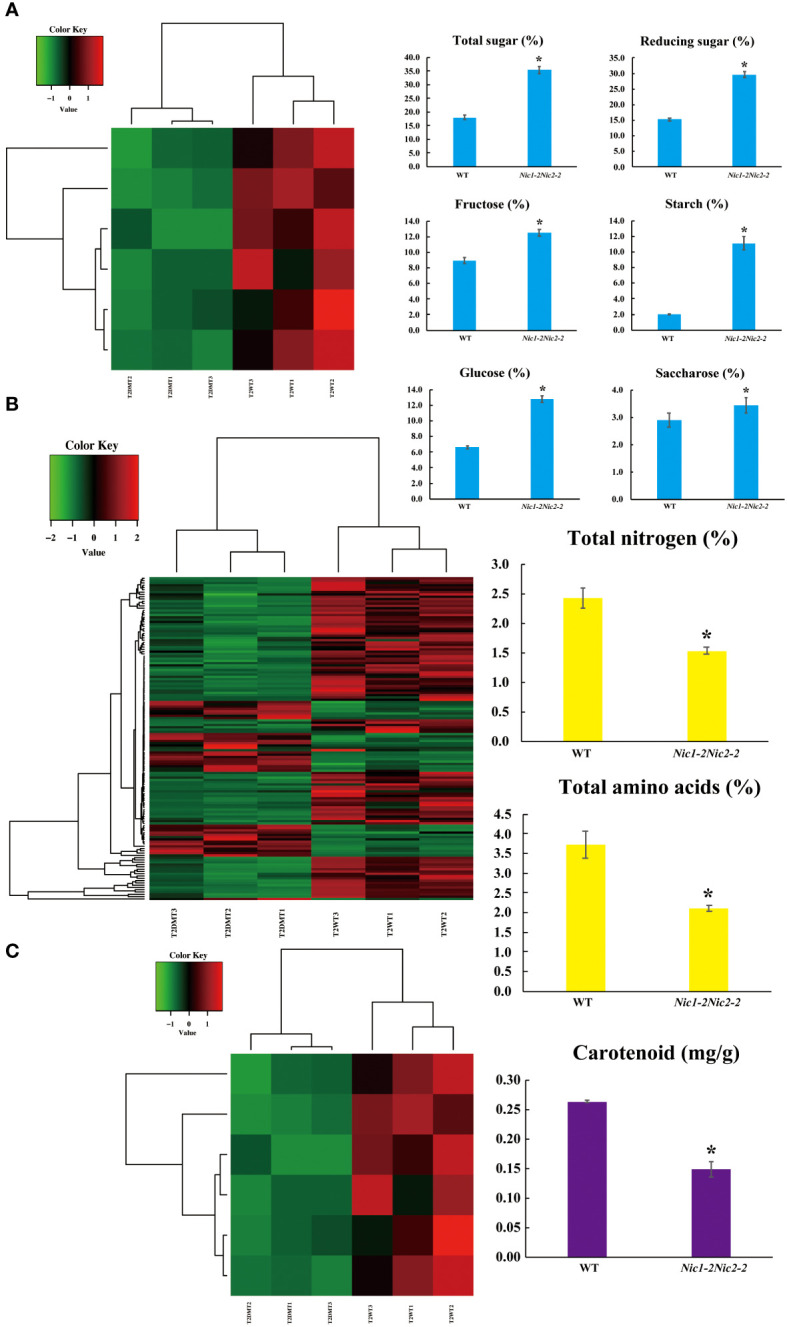
Correlation analysis between the DEGs identified and selected metabolites in the T2 stage transcriptomes. **(A)** Heatmap analysis of DEGs based on the GO terms related to carbohydrate metabolic process in the T2 transcriptomes. **(B)** Heatmap analysis of DEGs based on the GO terms involve in nitrogen related metabolic process and/or cellular amino acid metabolic process in the T2 transcriptomes. **(C)** Heatmap analysis of DEGs based on the GO terms related to carotenoid dioxygenase activity in the T2 transcriptomes. The red color denotes the highly expressed up-regulated DEGs, and the green color denotes down-regulated DEGs with lower expression levels. The gradation from red to green represents the transition from large to small values of a FPKM normalized log_2_ transformed counts. The sample names are indicated below each column. DEGs are defined by different colors, with the normalized expression levels employing a color gradient from low (green) to high (red). Metabolic profile of six carbohydrate compounds (blue color), total nitrogen and total amino acid (yellow color), and carotenoid (purple color) in the flue-cured leaves of the wild-type control and *Nic1-2Nic2-2* transgenic plants. Data represent means ± standard deviation (SD) of three replicates. *P<0.05(n=3), by Student’s t-test.

Carbon-nitrogen interactions between the shoots and roots, transported by the xylem and phloem, play a crucial role in secondary metabolism in tobacco. The reduction of photosynthetic carbon metabolism leads to a decreased nitrate assimilation, amino acid levels, as well as nicotine accumulation (Matt et al., 2002). Additionally, topping also induces significant changes in carbohydrate metabolism (e.g., starch metabolism, glycolysis/gluconeogenesis) and signal transduction in tobacco axillary shoot growth (Wang et al., 2018). In our case, we found a defect in nicotine synthesis leads to a profound shift in the balance between carbon -nitrogen metabolism and secondary metabolic consequences in nicotine deficient condition (**Figure 8**). Notably, we identified a substantial increase in KEGG pathways related to galactose metabolism (sly04075), amino sugar and nucleotide sugar metabolism (sly00940), carbon metabolism (ko01200), and starch and sucrose metabolism (sly00592) (**Figure 6**). These findings are also supported by the significant increase in six carbohydrate compounds as well as decrease in total nitrogen and total amino acid contents in low-nicotine leaves (**Figures 8A, B**). Interestingly, this alteration in carbon and nitrogen metabolism appears to activate a range of plant specialized metabolic pathways, likely driven by these newly identified TFs, including betalain biosynthesis, indole alkaloid biosynthesis, isoquinoline alkaloid biosynthesis, phenylpropanoid metabolism, and sequiterpenoid and triterpenoid biosynthesis (**Figure 5**). Conversely, the knockdown of *NtERF189/199* in *N. tabacum* appears to result in decreased nitrogen absorption and assimilation, as evidenced by reduced total nitrogen accumulation and the downregulation of amino acid metabolism pathways, such as valine, leucine, and isoleucine biosynthesis, ascorbate and aldarate metabolism, cysteine and methionine metabolism, and arginine and proline metabolism (**Figure 6**, **Supplementary Table 7**). Taken together, these results support the notion that the inhibition of nicotine synthesis, a nitrogen-rich secondary metabolite in tobacco leaves, triggers a broad activation of photosynthetic carbon metabolism and a reduction in nitrogen related metabolism, leading to significant changes in metabolic outcomes.

Nicotine is recognized as a qualitative resistance trait with a substantial impact on defending against pathogen attacks and herbivores. In contrast to predominant loci, which have large effects on resistance, studies on quantitative resistance, associated with small-effect loci responding to pathogen attacks, have been focused on specific defense-related outputs, such as reinforcing the cell wall or biosynthesis of defense compounds (Corwin and Kliebenstein, 2017). The successful identification of most causal genes for quantitative resistance often relies on our understanding of the known components of the plant innate immune system, which can be divided into three stages: pathogen perception, signal transduction, and defense response. In line with this model, our transcriptomic analysis revealed that a substantial number of genes associated with these three aspects are downregulated in the absence of nicotine. For instance, many causal genes involved in the pathogen perception process, such as calmodulin and calmodulin-like proteins, receptor-like protein kinase proteins, and plant U-box proteins, have been identified. The second-largest group of causal loci includes potential transcription factors and secondary metabolite enzymes, which are associated with signal transduction. This group encompasses *ERF*, *bHLH*, *WRKY*, *GRAS*, as well as genes related to downstream defense mechanisms, such as xyloglucan endotransglucosylase hydrolases, plant polyphenol oxidase, and 9-*cis*-epoxycarotenoid dioxygenase (**Figures 4**–**6**). Many of these identified genes have homologous counterparts in other plant species that function in response to both biotic and abiotic stresses. This observation further supports the idea that these identified genes likely include many causal genes associated with small-effect quantitative resistance in tobacco. The collective downregulation of these genes in the absence of nicotine suggests a compromised defense response in the low-nicotine condition, making the plant more vulnerable to various stressors.

In this study, we have conducted a comprehensive comparative transcriptomic analysis to explore the biological consequences of the loss of nicotine biosynthesis in tobacco across various growth periods. The results presented here contribute to a broader understanding of the regulatory role of nicotine biosynthesis in the entire primary and secondary metabolic networks. Furthermore, our findings shed light on the tight link between qualitative resistance (nicotine biosynthesis) and quantitative resistances in tobacco. Specifically, we have demonstrated that the disruption of nicotine synthesis leads to a significant decrease in the expression of genes involved in responding to diverse abiotic and biotic stresses, as well as herbivore resistance. The candidate genes we have identified, which play roles in the plant innate immunity system, hold the potential to serve as causal genes for quantitative resistance. Their molecular functions will be a subject of further investigation in future studies. This research not only deepens our understanding of the intricate interplay between nicotine biosynthesis and plant defense mechanisms but also opens up new avenues for exploring the molecular basis of quantitative resistance in tobacco.

## Data availability statement

The data presented in this study are deposited in the National Genomics Data Center (NGDC) repository, accession number CRA014456 (https://bigd.big.ac.cn/gsa/browse/CRA014456).

## Author contributions

XS: Data curation, Funding acquisition, Writing – review & editing. ZS: Data curation, Methodology, Writing – original draft, Writing – review & editing. RW: Data curation, Writing – original draft. HZ: Data curation, Writing – review & editing. ZT: Data curation, Writing – review & editing. CY: Data curation, Writing – review & editing. YL: Data curation, Writing – review & editing. CH: Data curation, Writing – review & editing. LZ: Data curation, Writing – review & editing. YW: Methodology, Writing – review & editing. YD: Methodology, Writing – review & editing.
