# Supplementary figures and images for: Comparative transcriptome analysis reveals nicotine metabolism is a critical component for enhancing stress response intensity of innate immunity system in tobacco
Source: Front Plant Sci. 2024 Mar 21;15:1338169. doi: 10.3389/fpls.2024.1338169 (PMC11003474; doi:10.3389/fpls.2024.1338169)

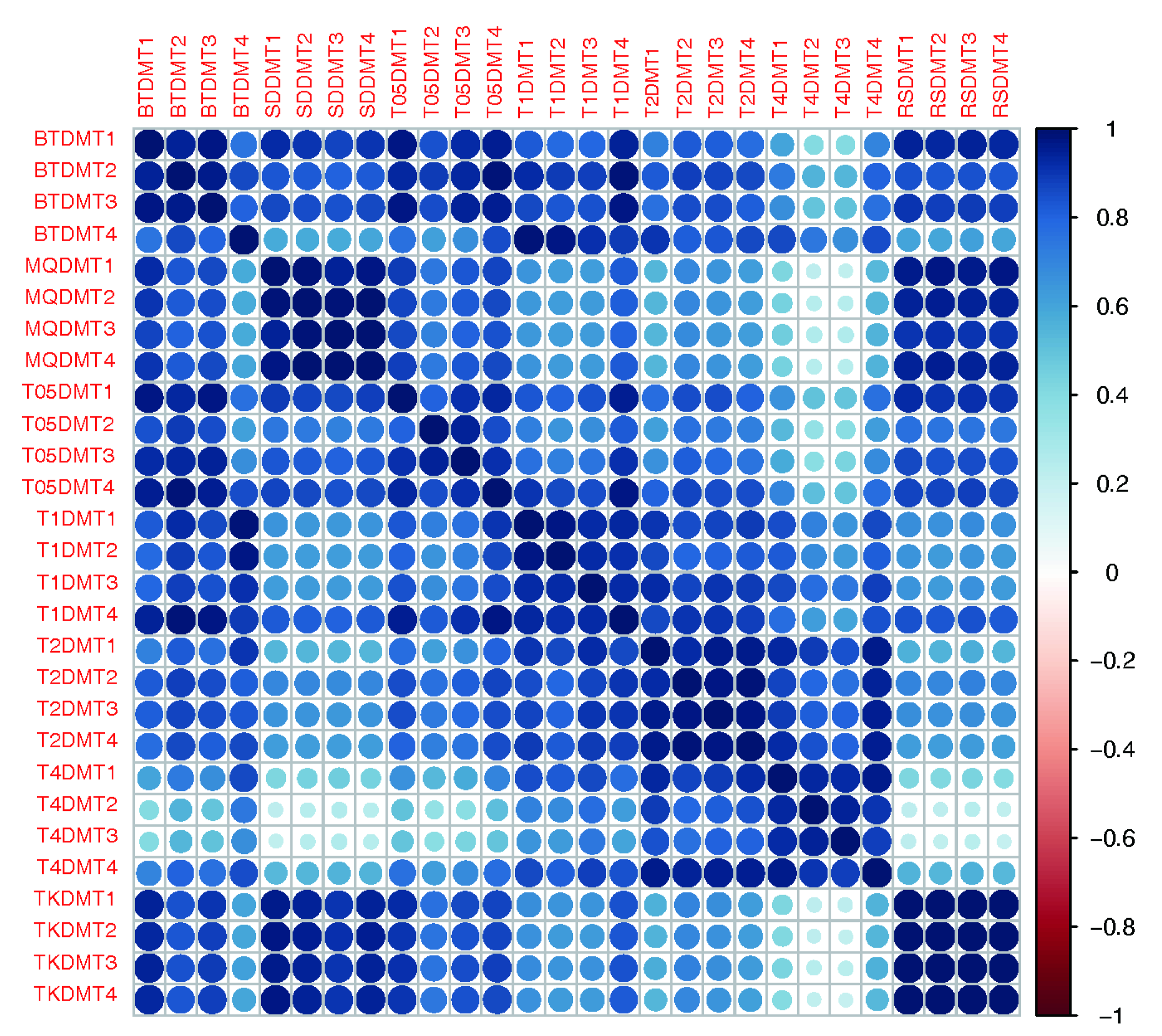

Supplement: Supplementary file 7 [file Image_1.tif]

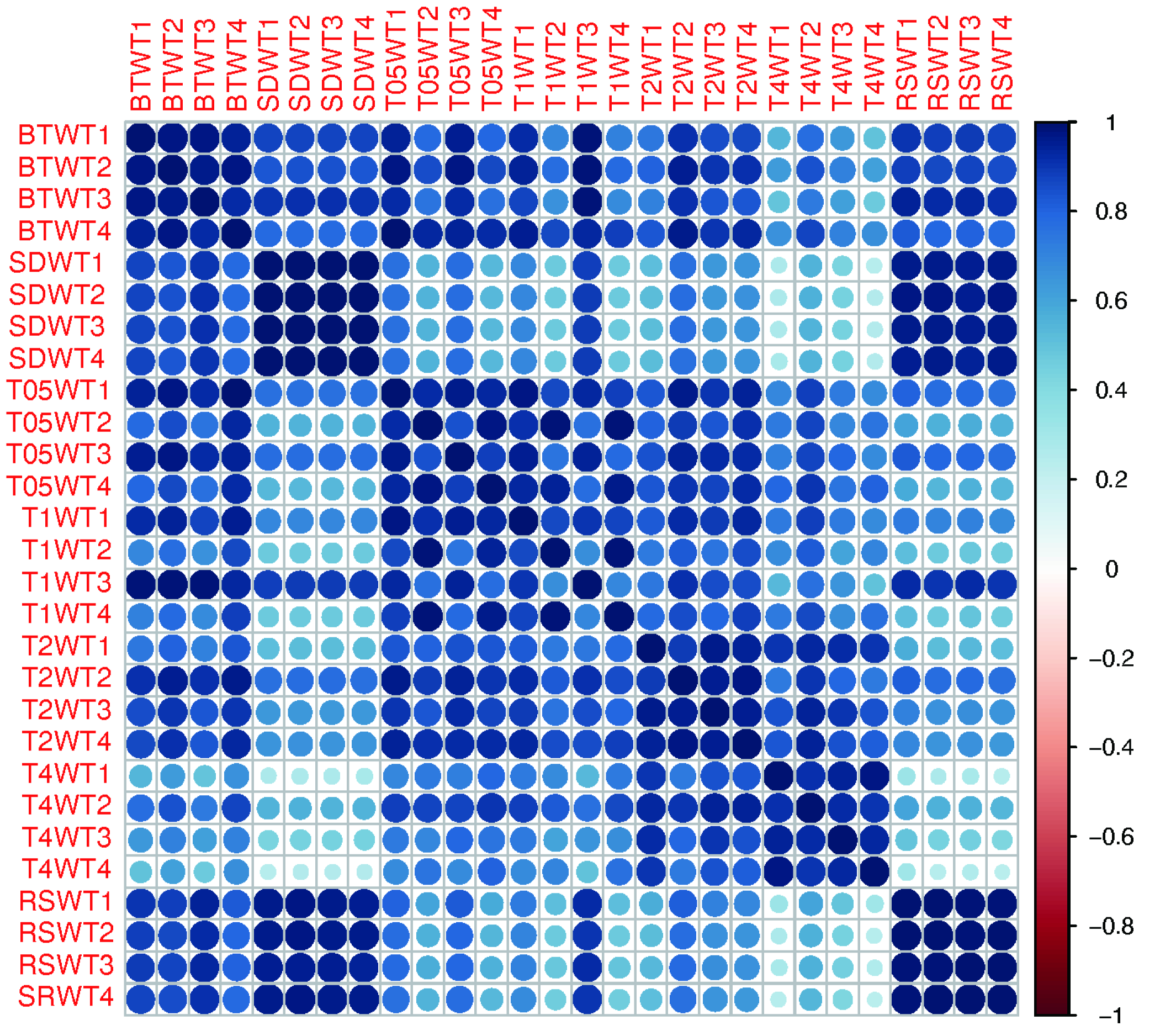

Supplement: Supplementary file 8 [file Image_2.tif]
